# A Rare Problem of School Work

**Published:** 1914-04-18

**Authors:** 


### NERVOUS DISEASES IN SCHOOL CHILDREN.

### A Rare Problem of School Work.

With the exception of epilepsy, nervous disease in school children is not at all frequently met with. The reason is, of course, that children in which the early signs of such disease show themselves are taken by their parents to the family or hospital doctor and receive prompt treatment. Only in a relatively few cases are they left for the school doctor to discover. At the routine examination the nervous system is considered in common with the other body systems. The examination can be easily and rapidly made, the main points to be attended to being the condition of the reflexes, the eyes, and the speech. Inco-ordination is common enough; chorea, for example, is quite a common disease among school children, though here again it is not met in its graver stages very frequently, since the parents observe the twitching or tremor and take the child for treatment.

When there is reason to suspect a nervous lesion the examination should be thorough and strict. The general condition of the child is noted; his carriage of body when standing erect or sitting, his gait, and his voluntary motions are observed. When the arm is stretched out and the fingers spread apart any slight tremor is readily distinguishable; a more reliable sign of inco-ordination is that known as Quinquad's phenomenon, which consists in the fine vibratory tremor communicated to the observer's palm when the child places the tips of his fingers against it and steadily presses upon it. This sign was originally stated to be indicative of alcoholic inco-ordination in adults; it is, however, met with in nearly all cases where there is lack of muscle tonus and inco-ordination, and is frequent in children with slight chorea. Any twitching or spasm of the face should be noted, and an attempt should be made to elicit nystagmus. This latter sign is by no means uncommon in children who have otherwise no symptom of nervous disease; although these children are apparently normal in all other respects they should be followed up and examined from time to time. The knee, ankle, and elbow jerks should be tested, and also the strength of the muscles. It is not always possible to make a close examination of the fundus of the eye, but where such an examination can be made it must not be omitted. On the whole, however, it is the gross signs that can alone be investigated; for a more searching examination the child must be remanded for further examination either at the clinic or at some hospital.

Obvious paralysis, usually the result of infantile hemiplegia or anterior poliomyelitis, is easily "spotted"; so too is chorea. The only difficulty is to differentiate the latter from habit spasm and spasmophilia. These are more common in infants than in pupils in the senior classes, though well-marked cases of habit spasm occur in the higher standards. In habit spasm the movements are not entirely involuntary; the child can be made to control them if his attention is directed to the movement, but as soon as he forgets the twitching is repeated. In chorea the movements are involuntary and cannot be controlled; in addition there are usually subsidiary signs, such as cardiac murmurs, anæmia, tremor, and headache. In spasmophilia, which may mimic chorea, the children are usually young infants; there is some history of colic or spasm of the involuntary muscles —tenesmus and pains simulating intussusception are not uncommon—and the mother will usually inform the doctor that the child grinds its teeth and is restless at night. Many of these cases are not true cases of spasmophilia, but are caused by worms. In these a quassia injection or the regular administration of petroleum emulsion usually works wonders, and it is as well to make the suggestion that the child has worms in every case. We now know that thread-worms elaborate a poison which specially acts upon the nervous system, raises the blood pressure, and leads to disturbance of the digestion. Recent investigations by German authors have shown that the importance of helminthic infection can scarcely be over-estimated in children; probably a large number of debilitated and so-called "pretubercular" children suffer from worms alone and will readily respond to treatment instituted for the particular disease.

Epilepsy is perhaps the most common nervous disease which the doctor will find. In only a few cases is it of the Jacksonian type; most cases are true idiopathic epilepsy. Unfortunately the minor degrees are not at all easy to demonstrate; cases of *petit mal*, which reveal themselves in slight signs, usually escape observation. The master perhaps brings a boy or girl with the complaint that he or she is inattentive in class or suffers from mind-wandering. Such children should be carefully examined, although the examination does not generally give conclusive evidence to warrant a diagnosis of epilepsy. *Grand mal* cases are much easier; the only question, in young children, is whether the convulsions are epileptic or caused by worms or some other one of the manifold causes of eclampsia in young children. Observation and watching the patient—who should, of course, have a follow-up card made out and seen every term—will correct any mistake made at the first examination. Where either *petit mal* or *grand mal* has been conclusively shown to exist it is better that the child should go to a special school. This is not always possible, but it is advisable to press the matter, since the epileptic child is a most disturbing factor in the ordinary school for various reasons. Treatment is wholly unsatisfactory; bromides should be avoided wherever possible; unfortunately they are the only drug we possess that can be relied upon to stop the fits. The dietetic treatment, recently vaunted in some quarters, has not proved of any value. Much attention is necessary in after life, and the question of the employment of the epileptic child is one of the problems which the school doctor will have to face. Where the child is of markedly low mental grade, the only resource is to get him admitted to an epileptic colony.

A few rare nervous diseases, such as Friedreich's ataxia, varieties of the dystrophies, and late paralysis after diphtheria, are sometimes met with; these need no special comment, since they are usually easy to distinguish, and the only possible treatment is prompt transference to a hospital where in-patient treatment can be given. A number of neuroses may cause some trouble; of these nervous aphonia is not infrequent; it is sometimes found in weak children after tuberculin injections.

